# Eukaryotic-Like Virus Budding in *Archaea*

**DOI:** 10.1128/mBio.01439-16

**Published:** 2016-09-13

**Authors:** Emmanuelle R. J. Quemin, Petr Chlanda, Martin Sachse, Patrick Forterre, David Prangishvili, Mart Krupovic

**Affiliations:** a Unité de Biologie Moléculaire du Gène chez les Extrêmophiles, Département de Microbiologie, Institut Pasteur, Paris, France; b Section on Integrative Biophysics, Eunice Kennedy Shriver National Institute of Child Health and Human Development, National Institutes of Health, Bethesda, Maryland, USA; c Ultrapole, Institut Pasteur, Paris, France

## Abstract

Similar to many eukaryotic viruses (and unlike bacteriophages), viruses infecting archaea are often encased in lipid-containing envelopes. However, the mechanisms of their morphogenesis and egress remain unexplored. Here, we used dual-axis electron tomography (ET) to characterize the morphogenesis of Sulfolobus spindle-shaped virus 1 (SSV1), the prototype of the family *Fuselloviridae* and representative of the most abundant archaea-specific group of viruses. Our results show that SSV1 assembly and egress are concomitant and occur at the cellular cytoplasmic membrane via a process highly reminiscent of the budding of enveloped viruses that infect eukaryotes. The viral nucleoprotein complexes are extruded in the form of previously unknown rod-shaped intermediate structures which have an envelope continuous with the host membrane. Further maturation into characteristic spindle-shaped virions takes place while virions remain attached to the cell surface. Our data also revealed the formation of constricted ring-like structures which resemble the budding necks observed prior to the ESCRT machinery-mediated membrane scission during egress of various enveloped viruses of eukaryotes. Collectively, we provide evidence that archaeal spindle-shaped viruses contain a lipid envelope acquired upon budding of the viral nucleoprotein complex through the host cytoplasmic membrane. The proposed model bears a clear resemblance to the egress strategy employed by enveloped eukaryotic viruses and raises important questions as to how the archaeal single-layered membrane composed of tetraether lipids can undergo scission.

## IMPORTANCE

The replication of enveloped viruses has been extensively studied in eukaryotes but has remained unexplored for enveloped viruses infecting bacteria and archaea. Here, we provide a sequential view on the assembly and egress of SSV1, a prototypic archaeal virus. The observed process is highly similar to the budding of eukaryotic enveloped viruses, including human immunodeficiency virus, influenza virus, and Ebola virus. The present study is the first to characterize such a phenomenon in archaeal cells, showing that membrane budding is not an exclusive feature of eukaryotic viruses. Our results provide significant insights into the biogenesis and architecture of unique, spindle-shaped virions that infect archaea. Furthermore, our findings open doors for future inquiries into (i) the evolution of the virus budding process, (ii) mechanistic details of virus-mediated membrane scission in *Archaea*, and (iii) elucidation of virus- and host-encoded molecular players responsible for archaeal membrane and surface remodeling.

## Observation

Enveloped viruses of eukaryotes, including important human pathogens, such as human immunodeficiency virus, influenza virus, or Ebola virus, typically escape their host cells via budding through cellular membranes, whereby they acquire the lipid-containing envelope. Similar to many eukaryotic viruses (and unlike bacteriophages), viruses infecting archaea often contain envelopes and host-derived lipids (1). However, the ways of their morphogenesis and egress remain largely unexplored. As a model for studies on the release of lipid-containing viruses of *Archaea*, we chose Sulfolobus spindle-shaped virus 1 (SSV1), the prototypic member of the family *Fuselloviridae*, which represents one of the most abundant and widely distributed archaea-specific groups of viruses (2). The SSV1 virions are composed of four virus-encoded proteins (VP1 to -4) and one host-encoded chromatin protein (Sso7d), which together with host-derived dibiphytanyl glycerol tetraether lipids enclose a circular double-stranded DNA (dsDNA) genome of 15.4 kb (3, 4). The structure of the SSV1 virion has been recently investigated by using cryo-electron microscopy and image reconstruction; however, the obtained low-resolution (~32 Å) map offered no insight on the existence of an envelope in the virion (5).

The assembly and release of SSV1 virions were studied using dual-axis electron tomography (ET) of lysogenic *Sulfolobus shibatae* B12 host cells. SSV1 production was induced by UV irradiation (3, 6), allowing synchronization of the virus replication cycle. The increase in the number of SSV1 plaque forming units (PFU) in the culture of UV-irradiated *S. shibatae* cells was concomitant with a decrease in the number of colony forming units (CFU), indicating loss of cell viability (see Fig. S1A and B in the supplemental material). Notably, following 24 h of growth, the SSV1 titer also increased in the nonirradiated control culture as it approached the stationary phase, likely due to spontaneous virus induction by starvation-associated stress. For analysis, samples were retrieved from the *S. shibatae* cultures at 0, 3, 6, 9, 12, and 24 h post-UV irradiation (hpi) and vitrified by high-pressure freezing. Two cell phenotypes were observed: (i) regular cells with an electron-dense cytoplasm and intact cytoplasmic membrane (see Fig. S1C and F) and (ii) “empty” cells, seemingly devoid of the cytoplasmic content (see Fig. S1D). In the control culture, the “empty” cells represented 4% of the population, whereas in the virus-induced culture their proportion increased to up to 50% (see Fig. S1E). Closer examination of the “empty” cells revealed severe rupture of the cytoplasmic membrane (see Fig. S1G and H), suggesting cell death, consistent with the decrease in the number of CFU (see Fig. S1B).

**FIG 1:**
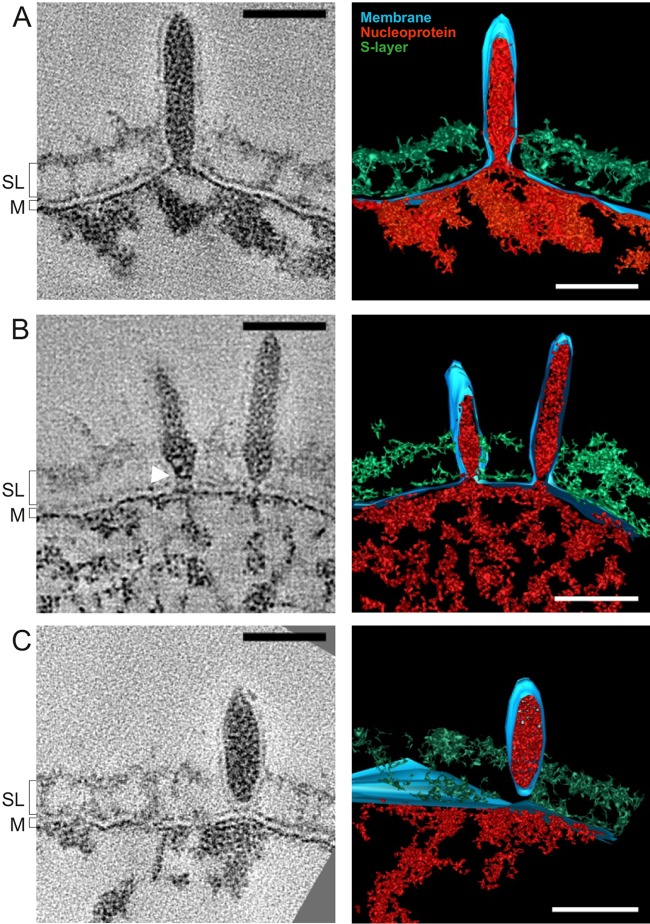
Different stages of SSV1 budding. (A to C) Slices through tomograms (left) and volume segmentations (right) showing concomitant assembly and release of SSV1 virions (see Videos S1 and S2 in the supplemental material). The white arrowhead marks an electron density presumed to be a ring-like structure. Red, putative nucleoprotein; blue, lipid membrane (M); green, S-layer (SL). Scale bars, 50 nm.

SSV1 virions were detected at the surface of the *S. shibatae* cells as early as 3 hpi (Fig. 1). Preformed capsids or discernible virion intermediates were not observed in the cytoplasm. Instead, there was a bulge in the cellular membrane caused by the electron-dense material (Fig. 1), potentially corresponding to the previously characterized SSV1 nucleoprotein composed of the viral dsDNA complexed with proteins VP2 and Sso7d (3, 4). Viral nucleoprotein was enclosed by an envelope derived from the cytoplasmic membrane. This observation suggests that SSV1 assembly, as seen with the assembly of some eukaryotic viruses (e.g., influenza virus [7]), commences directly at the host cell membrane. Some virions were attached to the host cell surface, with their envelope continuous with the cell membrane (Fig. 1A). Notably, the membrane in the virions appeared thinner (3.6 ± 0.5 nm [mean ± standard deviation]; *n* = 35) than that in the host cell (5.0 ± 0.2 nm; *n* = 20). To further characterize the features of the host and SSV1 membranes, we compared the densities of the corresponding membrane layers (see Fig. S2A to C in the supplemental material). The generated line plots showed two clearly distinguishable surfaces of the cellular membrane, with the outer layer (OL) continuous with the OL of the SSV1 membrane. The density corresponding to the inner layer (IL) of the viral membrane was indistinguishable from that of the viral nucleoprotein core, although the IL of the host membrane was readily discernible (see Fig. S2A to C). To verify whether the membrane is retained after virion budding and is an integral part of the virion, we performed thin sectioning of resin-embedded purified viral particles. Transmission electron microscopy analysis revealed the presence of the envelope surrounding the electron-dense core (see Fig. S2D), in accordance with the presence of host-derived lipids in the highly purified SSV1 virions (3).

**FIG 2:**
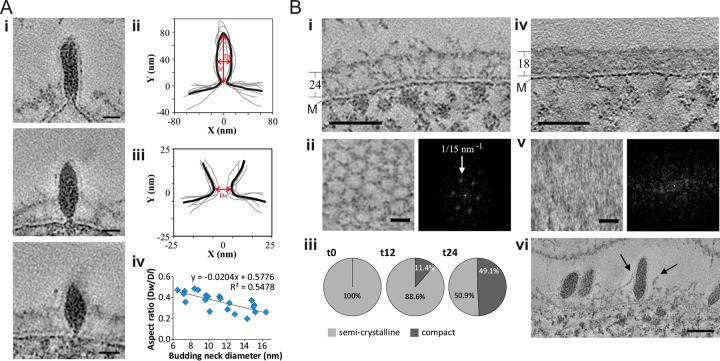
Membrane constriction and reorganization of the host S-layer. (A, panel i) Three slices through tomograms showing virions attached to the cell surface (see Videos S3 and S4 in the supplemental material). Scale bars, 20 nm. (ii and iii) Aligned and averaged (bold line) 2D contours of 7 virions (ii) and their budding necks (iii). Dw, average width; Dl, average length; Dn, neck diameter. (iv) Virion budding neck diameters, plotted against the aspect ratio of the virions. (B) Slices through tomograms of *S. shibatae*, showing a semicrystalline S-layer (i) (see Video S5 in the supplemental material) and compact S-layers (iv) (see Video S6 in the supplemental material). S-layer thickness and the membrane (M) are indicated. (ii and v) Top views (left) and power spectra (right) of semicrystalline (ii) and compact (v) S-layers. (iii) Relative distribution of S-layer types at 0 (*n* = 195), 12 (*n* = 386), and 24 (*n* = 122) hpi. Only cells with the electron-dense cytoplasm were considered in calculating the S-layer phenotypes. (vi) Slice through a tomogram showing the S-layer rupture (arrows) during virion release. Scale bars, 50 nm (i and iv), 20 nm (ii and v), and 50 nm (vi).

The late stages of virion morphogenesis were linked with virion budding and release. At 12 hpi, 26% of the virions (*n* = 27) showed a constricted budding neck of varied diameter at the trailing end of the virion bud (Fig. 1B and 2A, panel i). We aligned two-dimensional (2D) contours representing the central longitudinal tomographic slices of the 7 virions and their budding necks to calculate the average profile of the budding virion and its neck (Fig. 2A, panels ii and iii). The average diameter of the budding neck was 11.5 ± 3.8 nm. To further confirm that the virions were constricted at the trailing end, we calculated the radius (*R*) of the osculating circle and obtained the meridian curvature (*J*) of the constriction, as 1/*R* (see the equation below). Budding necks were often asymmetrical along the longest axis of the virion (Fig. 2A), with meridian curvature ranging from 0.12 to 0.53 nm^−1^, consistent with a general geometry of a budding neck.

The majority of SSV1 virions (74%), after separation from the cell membrane (Fig. 1C), remained attached to the cell surface, presumably trapped in the protein surface (S) layer, which coats *Sulfolobus* cells (8). ET analysis showed that the S-layer in virus-induced *S. shibatae* cells undergoes a significant morphological transformation. Prior to virus induction, the vast majority of cells (*n* = 195) contained the semicrystalline, porous S-layer (thickness of 24 ± 2 nm; *n* = 10) with a hexagonal pattern and vertex-to-vertex distance of ~15 nm, characteristic of the members of the order *Sulfolobales* (9) (Fig. 2B, panels i to iii). Following induction, however, by 24 hpi ~49% of the cell population (*n* = 122) exhibited the more compact S-layer (thickness of 18 ± 4 nm; *n* = 10) that was devoid of the hexagonal pattern (Fig. 2B, panels iii to v). The hexagonal pores in the semicrystalline S-layer of *S. shibatae* cells were smaller (~15 nm) (Fig. 2B) than the smallest measured diameter of the SSV1 budding virions (~17 nm), indicating that the virion must disrupt the S-layer in order to get released. The less-structured, thinner S-layer might present a more permissive barrier for the budding virus. Indeed, we observed ruptures in the S-layer at the points where SSV1 particles were localized (Fig. 2B, panel vi), suggesting that the virus might locally disrupt the S-layer upon exit. Notably, some SSV1 virions (30%; *n* = 27 at 12 hpi) contained discernible fibers on the leading tip of the viral particle (see Fig. S2E in the supplemental material); it remains unclear, however, whether these fibers represent the characteristic terminal appendages located at one of the pointed ends of the purified SSV1 virions (3, 5, 10).

SSV1 virions associated with the cell surface were either spindle shaped or rod shaped, suggesting that virions trapped in the S-layer are undergoing maturation through shape transition. The average virion width was 28 ± 4 (*n* = 11), 23 ± 4 nm (*n* = 27), and 27 ± 4 nm (*n* = 21) at 9, 12, and 24 hpi, respectively. To distinguish between spindle- and rod-shaped virions, we measured the length (Dl) and width (Dw) of the virions and calculated the corresponding aspect ratio, R_a_ (Dw/Dl). The R_a_ at 9, 12, and 24 hpi was 0.37, 0.25, and 0.39, respectively, indicating that both rod- and spindle-shaped virions are present on the cell surface throughout infection. Remarkably, the virion aspect ratio was directly proportional to the budding neck diameter (Fig. 2A, panel iv). The spindle-shaped virions with a high aspect ratio (~0.5) had narrower budding necks, as opposed to rod-shaped virions, which exhibited rather large budding neck diameters (Fig. 2A, panel i). This observation indicated that the virion shape transition (maturation) is concomitant with budding neck constriction. Whether constriction is driven by virion maturation and whether cellular factors play a role in this process remain to be investigated. To elucidate if the shape transition and neck constriction also correlate with membrane scission, we compared the aspect ratios of the virions with budding necks and those of virions after membrane scission. The normal-fitted distributions of the aspect ratios of both budding and detached virions were similar, with an average of 0.35 (see Fig. S3 in the supplemental material). Thus, both rod-shaped and spindle-shaped virions are able to undergo membrane scission. Interestingly, some budding necks (*n* = 4) contained a single electron-dense ring-like structure (Fig. 1B, white arrowhead), and in one case we observed a budding neck with two ring-like structures (see middle panel in Fig. 2A, panel i, middle; see also Fig. S2F in the supplemental material). The spacing between the rings was 5.6 nm, and they were tilted at an angle of 65° with respect to the direction of budding. Similar spacing has been reported in various helical ESCRT-III assemblies, which are the key component of the cellular membrane remodeling machinery hijacked by many eukaryotic enveloped viruses during budding (11). For instance, an interfilament spacing of 5.1 nm has been observed in the helical copolymer composed of human ESCRT-III subunits CHMP1B and IST1 (12). Importantly, hyperthermophilic archaea of the order Sulfolobales, including the host of SSV1, encode functional ESCRT-based machinery which plays a central role during cell division, as demonstrated by different approaches, including a recent cryo-ET study (13–16). Thus, membrane scission during SSV1 budding is more likely to be driven by cellular membrane remodeling machinery rather than by shape transition, whereas virion maturation can occur either while budding or following membrane scission.

Collectively, our results provide a sequential view on the assembly and egress of the archaeal virus SSV1. The SSV1 nucleoprotein complex is extruded through the cytoplasmic membrane in the form of an enveloped, tubular structure which subsequently assumes the spindle-shaped morphology of the mature virion (Fig. 1). Notably, some fuselloviruses, namely, members of the genus *Betafusellovirus*, have elongated rather than spindle-shaped virions in the native state (17); thus, maturation might not be universal for all members of the family. Our results indicate that SSV1 acquires the envelope by budding through the cytoplasmic membrane of the host, and the envelope is retained in the mature infectious virions. The final step in the budding process involves the formation of a budding neck, a structure observed prior to membrane scission in various enveloped viruses of eukaryotes (11). Once membrane scission has occurred, the viruses locally disrupt the S-layer and are released into the environment. The proposed model bears clear resemblance to the exit strategy employed by enveloped eukaryotic viruses. Notably, our results indicate that a single-layer archaeal membrane composed of tetraether lipids (3) can undergo scission. In the future, it will be important to elucidate which viral and cellular factors play a role in virus release and whether parallels between the budding of archaeal and eukaryotic viruses can be extended to the molecular level.

### Virus, archaeal strain, and growth conditions.

*Sulfolobus shibatae* B12 was used as a host for SSV1 (10). Virus induction was performed as described previously (3).

### Sample preparation for electron microscopy.

Cultures at 0, 3, 6, 9, 12, and 24 hpi were pelleted by low-speed centrifugation and resuspended in a minimal volume of growth medium; SSV1 virions were concentrated by polyethylene glycol precipitation as previously described (3) and further processed into capillaries. Cell pastes or capillaries were transferred into a lecithin-coated sample holder type A and frozen with a high-pressure freezing machine (HPM 010; Bal-Tec Products, Middlebury, CT). Following cryo-fixation, the samples were freeze-substituted with 0.5% glutaraldehyde (Electron Microscopy Sciences, Washington, PA, USA), 1% OsO_4_ (Merck Millipore, Germany), 0.2% uranyl acetate (Merck, Darmstadt, Germany), 2% H_2_O, and 4% methanol in acetone (Electron Microscopy Sciences, Washington, PA, USA) according to the following schedule: −90°C for 40 h, 5°C/h for 6 h, −60°C for 8 h, 5°C/h for 6 h, and −30°C for 8 h. The cells were rinsed three times in acetone and slowly infiltrated with Agar 100 epoxy resin (Agar Scientific, United Kingdom). After heat polymerization, 70-nm thin sections were cut with an Ultracut R microtome (Leica, Vienna, Austria) and collected on Formvar-coated copper grids (Electron Microscopy Sciences, Washington, PA, USA). Sections were poststained with 4% uranyl acetate for 45 min, followed by 5 min in Reynold’s lead citrate. The grids were viewed using a Tecnai T12 transmission electron microscope (FEI, OR, USA) operated at 120 kV.

### Electron tomography.

For electron tomography, embedded cells were cut into serial 200-nm-thick sections with an Ultracut R microtome (Leica, Vienna, Austria) and collected on Formvar-coated copper slot grids (Electron Microscopy Sciences, Washington, PA, USA). The sections were decorated with 10-nm protein A-gold particles (EMS, Hatfield, PA) on both sides of the section and poststained with 2% lead citrate in water. Single- or dual-axis electron tomography was performed in a Tecnai T20 transmission electron microscope (FEI, Eindhoven, Netherlands) operated at 200 kV and equipped with a K2 Summit camera (Gatan, Pleasanton, CA). Tomographic tilt ranges were collected using the SerialEM program (18), typically from +55° to −55° with an angular increment of 1° at nominal magnification of ×14,500 and pixel size of 0.259 nm. For tomogram reconstruction we used the weighted back-projection method, and the measurements were done in the IMOD software suite (19). The number of analyzed tomograms was 1, 1, 2, 4, 7, and 4 for samples vitrified at 0, 3, 6, 9, 12, and 24 h post-UV irradiation, respectively. Collectively, 61 budding events were recorded and analyzed.

The meridian curvature was calculated using the budding profiles, which were generated by outlining the membrane of the budding neck and exported to *x*-*y* coordinates. Each side of the budding neck profile was treated as a curve, which was fitted with a polynomial function with 2 terms within MatLab (MathWorks, MA, USA). This allowed calculation of the first and second derivatives of the fitted function and calculation of the curvature at each point of the curve (1/*R*) by an osculating circle method, where *R* was determined by the following equation:

$$R=\frac{{[1+{\left(\frac{dy}{dx}\right)}^{2}]}^{\frac{3}{2}}}{\left|\frac{d^{2}y}{dx^{2}}\right|}$$

## SUPPLEMENTAL MATERIAL

Video S1  Budding of a rod-shaped SSV1 virus. The video of a tomogram and rendering corresponds to that in Fig. 1A. Scale bar, 50 nm. Download Video S1, AVI file, 6.4 MB

Video S2  Spindle-shaped SSV1 virus, which has undergone membrane scission. The video shows a tomogram and rendering corresponding to that in Fig. 1C. Scale bar, 50 nm. Download Video S2, AVI file, 7.6 MB

Video S3  Budding SSV1 virus with a wide budding neck. The video of a tomogram corresponds to that shown in Fig. 2A, panel i (top). Scale bar, 20 nm. Download Video S3, AVI file, 1.9 MB

Video S4  Budding spindle-shaped SSV1 virus with narrow budding neck. The video shows a tomogram corresponding to that in Fig. 2A, panel i (bottom). Scale bar, 20 nm. Download Video S4, AVI file, 0.8 MB

Video S5  Semicrystalline S-layer. The video of a tomogram corresponds to that shown in Fig. 2B, panel i. Scale bar, 50 nm. Download Video S5, AVI file, 6 MB

Video S6  Compact S-layer. The video of a tomogram corresponds to that shown in Fig. 2B, panel iv. Scale bar, 50 nm. Download Video S6, AVI file, 11.3 MB

Video S7  An SSV1 budding virion with fibers on a terminal tip. The video corresponds to the tomogram shown in Fig. S2E. Scale bar, 20 nm. Download Video S7, AVI file, 1.1 MB

Figure S1  Changes in an *S. shibatae* population upon virus induction. (A) Optical densities of noninduced (circles) and induced (triangles) cultures, with a bar plot showing PFU titers for cell-free supernatants. Dark gray, nonirradiated cells; light gray, UV-irradiated (i.e., induced) cells. (B) Ratio of CFU versus the initial count at 0 hpi. Dark gray, noninduced control; light gray, UV-irradiated conditions. Error bars represent standard deviations from 3 independent experiments. (C to H) Phenotypic changes in the *S. shibatae* population upon virus induction. (C and D) Slices through tomograms of regular (C) and “empty” (D) cells. (E) Proportion of cellular phenotypes after virus induction. Black, regular cells; white, “empty” cells. (F to H) Close-up views on the envelopes of regular (F) and “empty” (G and H) cells, showing continuous and broken cytoplasmic membranes (black arrows), respectively. Scale bars, 50 nm. Download Figure S1, PDF file, 1.6 MB

Figure S2  Structural features of SSV1 virions. (A) A slice through a tomogram reconstructed using the simultaneous iterative reconstruction technique (SIRT), illustrating continuity between the membranes of the budding SSV1 and the host (white arrowheads). White rectangles indicate the areas shown in panel B. The membrane (M) with its outer layer (OL) and inner layer (IL) are indicated. The bottom image is identical to the image above, but with inverted contrast. Scale bars. 50 nm. (B) Membrane regions of SSV1 and the host cell. A membrane (M) with its outer layer (OL) and inner layer (IL) are indicated. The IL of the viral membrane shows a very similar electron density to the viral nucleoprotein core, which makes the two virtually indistinguishable. Thus, the presumed viral IL is indicated with a question mark. Scale bar, 10 nm. (C) Averaged linear density profiles of SSV1 (blue) and host cell membrane (red). Arrows point to the densities which can be attributed to the inner layer (IL) and outer layer (OL) surfaces of the membrane monolayer. The density of the host membrane inner layer is similar to the density of the SSV1 capsid, suggesting that the internal lipid layer might be embedded in the protein capsid. The density of the IL is approximately 1.2× higher than the density of the OL host membrane. The increased (1.6×) density of the host OL compared to the OL of SSV1 is likely caused by the proteinaceous S-layer. Dotted lines represent upper and lower boundaries of the standard deviations. (D) Representative electron micrograph of thin sections of resin-embedded SSV1, showing the presence of an envelope in the purified SSV1 virions. Scale bar, 200 nm. (E) Location of the terminal fibers on the leading tip of the viral particle. The slice through a tomogram shows fibers (arrow) on the budding virion. Scale bar, 20 nm. See also Video S7 in this supplemental material. (F) A slice through a tomogram (also shown in Fig. 2A, panel i, middle) reconstructed using SIRT revealed two electron-dense ring-like structures separated by 5.6 nm and tilted 65° with respect to the longitudinal axis of the budding virion. Scale bar, 20 nm (left image) or 5 nm (right image). Download Figure S2, PDF file, 2.1 MB

Figure S3  Comparison of aspect ratio distributions before and after SSV1 membrane scission. Histograms and normal-fitted probability density functions (PDF) of the aspect ratio distributions of virions before (budding data; *n* = 22) and after scission (released data; *n* = 37) are shown. Normal fitting was done using MatLab (MathWorks, MA, USA). Fitting parameters were as follows: for budding data, mu = 0.35 ± 0.03 and sigma = 0.095 ± 0.026; for released data, mu = 0.37 ± 0.02 and sigma = 0.097 ± 0.013. Download Figure S3, TIF file, 0.1 MB

## References

[1] Roine E, Bamford DH 2012 Lipids of archaeal viruses. Archaea 2012:384919. doi:10.1155/2012/384919. 23049284 PMC3461281

[2] Krupovic M, Quemin ER, Bamford DH, Forterre P, Prangishvili D 2014 Unification of the globally distributed spindle-shaped viruses of the Archaea. J Virol 88:2354–2358. doi:10.1128/JVI.02941-13. 24335300 PMC3911535

[3] Quemin ER, Pietilä MK, Oksanen HM, Forterre P, Rijpstra WI, Schouten S, Bamford DH, Prangishvili D, Krupovic M 2015 Sulfolobus spindle-shaped virus 1 contains glycosylated capsid proteins, a cellular chromatin protein, and host-derived lipids. J Virol 89:11681–11691. doi:10.1128/JVI.02270-15. 26355093 PMC4645638

[4] Reiter W, Palm P, Henschen A, Lottspeich F, Zillig W, Grampp B 1987 Identification and characterization of the genes encoding three structural proteins of the Sulfolobus virus-like particle SSV1. Mol Gen Genet 206:144–153. doi:10.1007/BF00326550.

[5] Stedman KM, DeYoung M, Saha M, Sherman MB, Morais MC 2015 Structural insights into the architecture of the hyperthermophilic fusellovirus SSV1. Virology 474:105–109. doi:10.1016/j.virol.2014.10.014. 25463608

[6] Schleper C, Kubo K, Zillig W 1992 The particle SSV1 from the extremely thermophilic archaeon Sulfolobus is a virus: demonstration of infectivity and of transfection with viral DNA. Proc Natl Acad Sci U S A 89:7645–7649. doi:10.1073/pnas.89.16.7645. 1502176 PMC49767

[7] Chlanda P, Zimmerberg J 2016 Protein-lipid interactions critical to replication of the influenza A virus during infection. FEBS Lett 590:1940–1954. doi:10.1002/1873-3468.12118. 26921878 PMC5007136

[8] Albers SV, Meyer BH 2011 The archaeal cell envelope. Nat Rev Microbiol 9:414–426. doi:10.1038/nrmicro2576. 21572458

[9] Veith A, Klingl A, Zolghadr B, Lauber K, Mentele R, Lottspeich F, Rachel R, Albers SV, Kletzin A 2009 Acidianus, Sulfolobus and Metallosphaera surface layers: structure, composition and gene expression. Mol Microbiol 73:58–72. doi:10.1111/j.1365-2958.2009.06746.x. 19522740

[10] Martin A, Yeats S, Janekovic D, Reiter WD, Aicher W, Zillig W 1984 SAV 1, a temperate uv-inducible DNA virus-like particle from the archaebacterium Sulfolobus acidocaldarius isolate B12. EMBO J 3:2165–2168. 16453555 10.1002/j.1460-2075.1984.tb02107.x PMC557659

[11] Hurley JH, Hanson PI 2010 Membrane budding and scission by the ESCRT machinery: it’s all in the neck. Nat Rev Mol Cell Biol 11:556–566. doi:10.1038/nrm2937. 20588296 PMC2922035

[12] McCullough J, Clippinger AK, Talledge N, Skowyra ML, Saunders MG, Naismith TV, Colf LA, Afonine P, Arthur C, Sundquist WI, Hanson PI, Frost A 2015 Structure and membrane remodeling activity of ESCRT-III helical polymers. Science 350:1548–1551. doi:10.1126/science.aad8305. 26634441 PMC4684769

[13] Dobro MJ, Samson RY, Yu Z, McCullough J, Ding HJ, Chong PL, Bell SD, Jensen GJ 2013 Electron cryotomography of ESCRT assemblies and dividing Sulfolobus cells suggests that spiraling filaments are involved in membrane scission. Mol Biol Cell 24:2319–2327. doi:10.1091/mbc.E12-11-0785. 23761076 PMC3727925

[14] Makarova KS, Yutin N, Bell SD, Koonin EV 2010 Evolution of diverse cell division and vesicle formation systems in Archaea. Nat Rev Microbiol 8:731–741. doi:10.1038/nrmicro2406. 20818414 PMC3293450

[15] Moriscot C, Gribaldo S, Jault JM, Krupovic M, Arnaud J, Jamin M, Schoehn G, Forterre P, Weissenhorn W, Renesto P 2011 Crenarchaeal CdvA forms double-helical filaments containing DNA and interacts with ESCRT-III-like CdvB. PLoS One 6:e21921. doi:10.1371/journal.pone.0021921. 21760923 PMC3132758

[16] Samson RY, Obita T, Freund SM, Williams RL, Bell SD 2008 A role for the ESCRT system in cell division in archaea. Science 322:1710–1713. doi:10.1126/science.1165322. 19008417 PMC4121953

[17] Redder P, Peng X, Brügger K, Shah SA, Roesch F, Greve B, She Q, Schleper C, Forterre P, Garrett RA, Prangishvili D 2009 Four newly isolated fuselloviruses from extreme geothermal environments reveal unusual morphologies and a possible interviral recombination mechanism. Environ Microbiol 11:2849–2862. doi:10.1111/j.1462-2920.2009.02009.x. 19638177

[18] Mastronarde DN 2005 Automated electron microscope tomography using robust prediction of specimen movements. J Struct Biol 152:36–51. doi:10.1016/j.jsb.2005.07.007. 16182563

[19] Kremer JR, Mastronarde DN, McIntosh JR 1996 Computer visualization of three-dimensional image data using IMOD. J Struct Biol 116:71–76. doi:10.1006/jsbi.1996.0013. 8742726

