# Supplementary figures and images for: Eukaryotic-Like Virus Budding in Archaea
Source: mBio. 2016 Sep 13;7(5):e01439-16. doi: 10.1128/mBio.01439-16 (PMC5021807; doi:10.1128/mBio.01439-16)

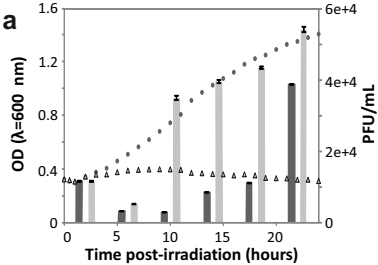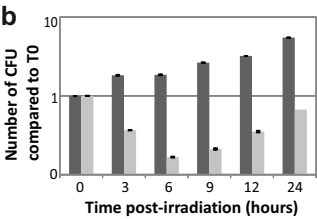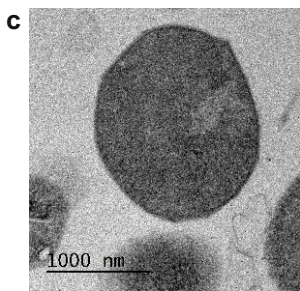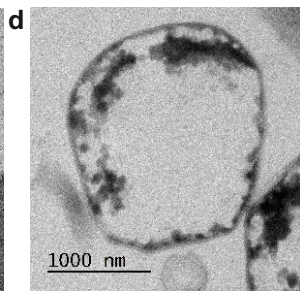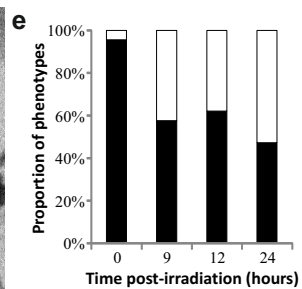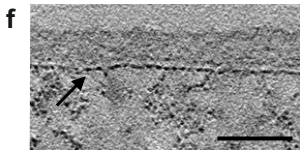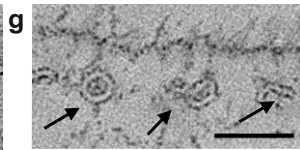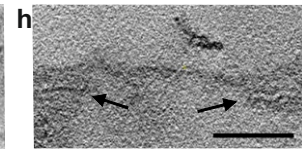

Supplement: Figure S1 — Changes in an S. shibatae population upon virus induction. (A) Optical densities of noninduced (circles) and induced (triangles) cultures, with a bar plot showing PFU titers for cell-free supernatants. Dark gray, nonirradiated cells; light gray, UV-irradiated (i.e., induced) cells. (B) Ratio of CFU versus the initial count at 0 hpi. Dark gray, noninduced control; light gray, UV-irradiated conditions. Error bars represent standard deviations from 3 independent experiments. (C to H) Phenotypic changes in the S. shibatae population upon virus induction. (C and D) Slices through tomograms of regular (C) and “empty” (D) cells. (E) Proportion of cellular phenotypes after virus induction. Black, regular cells; white, “empty” cells. (F to H) Close-up views on the envelopes of regular (F) and “empty” (G and H) cells, showing continuous and broken cytoplasmic membranes (black arrows), respectively. Scale bars, 50 nm. Download [file mbo004162977sf1.pdf]

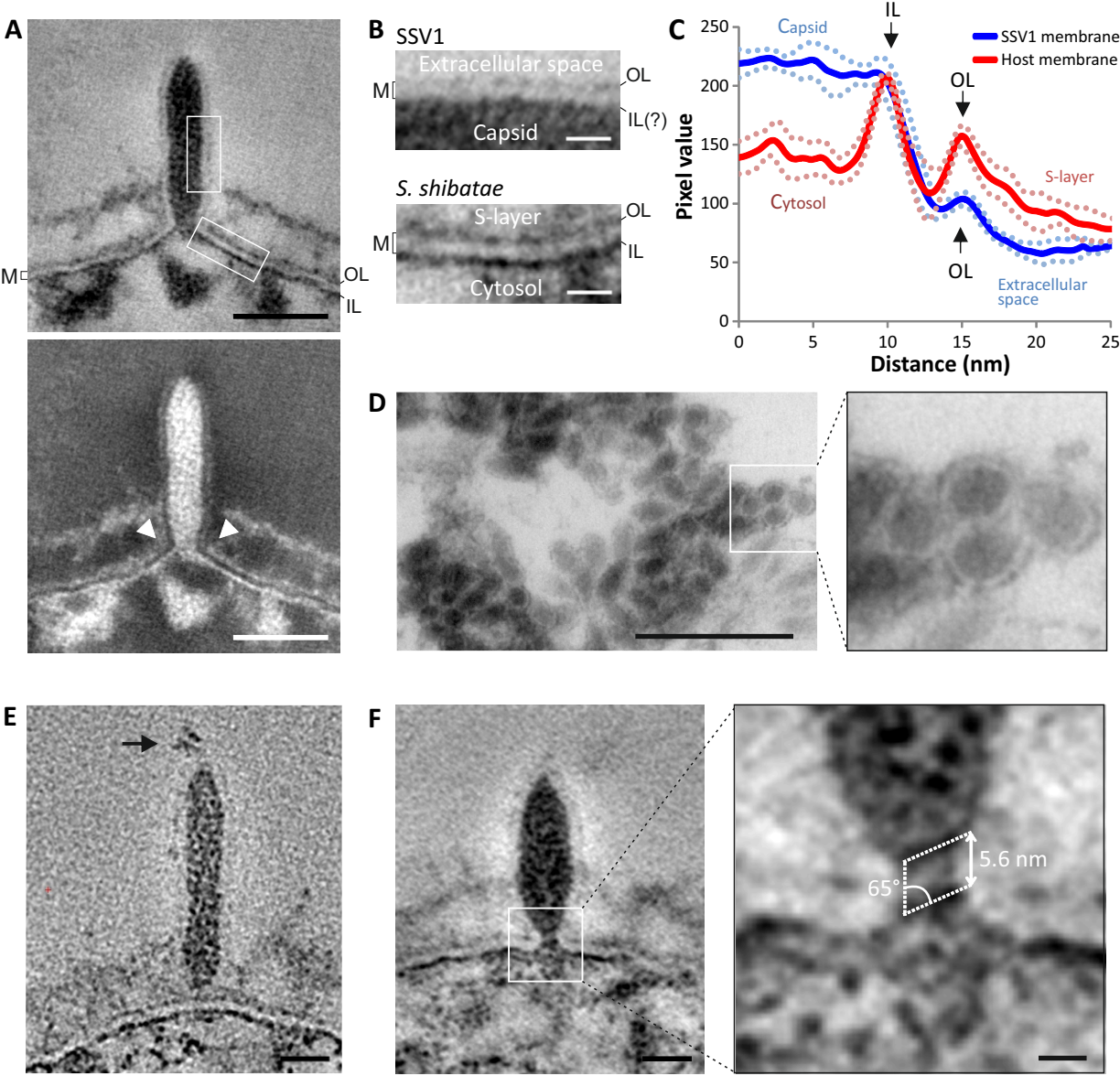

Supplement: Figure S2 — Structural features of SSV1 virions. (A) A slice through a tomogram reconstructed using the simultaneous iterative reconstruction technique (SIRT), illustrating continuity between the membranes of the budding SSV1 and the host (white arrowheads). White rectangles indicate the areas shown in panel B. The membrane (M) with its outer layer (OL) and inner layer (IL) are indicated. The bottom image is identical to the image above, but with inverted contrast. Scale bars. 50 nm. (B) Membrane regions of SSV1 and the host cell. A membrane (M) with its outer layer (OL) and inner layer (IL) are indicated. The IL of the viral membrane shows a very similar electron density to the viral nucleoprotein core, which makes the two virtually indistinguishable. Thus, the presumed viral IL is indicated with a question mark. Scale bar, 10 nm. (C) Averaged linear density profiles of SSV1 (blue) and host cell membrane (red). Arrows point to the densities which can be attributed to the inner layer (IL) and outer layer (OL) surfaces of the membrane monolayer. The density of the host membrane inner layer is similar to the density of the SSV1 capsid, suggesting that the internal lipid layer might be embedded in the protein capsid. The density of the IL is approximately 1.2× higher than the density of the OL host membrane. The increased (1.6×) density of the host OL compared to the OL of SSV1 is likely caused by the proteinaceous S-layer. Dotted lines represent upper and lower boundaries of the standard deviations. (D) Representative electron micrograph of thin sections of resin-embedded SSV1, showing the presence of an envelope in the purified SSV1 virions. Scale bar, 200 nm. (E) Location of the terminal fibers on the leading tip of the viral particle. The slice through a tomogram shows fibers (arrow) on the budding virion. Scale bar, 20 nm. See also Video S7 in this supplemental material. (F) A slice through a tomogram (also shown in Fig. 2A, panel i, middle) reconstructed using SIRT revealed [file mbo004162977sf2.pdf]

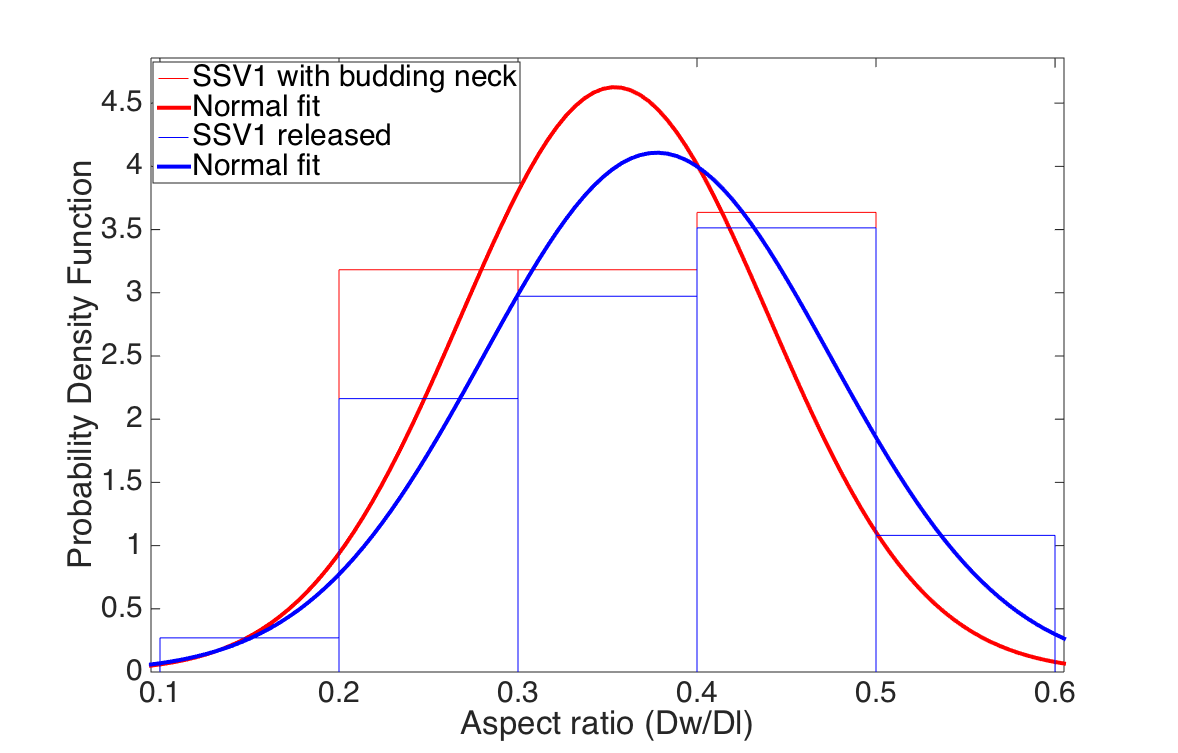

Supplement: Figure S3 — Comparison of aspect ratio distributions before and after SSV1 membrane scission. Histograms and normal-fitted probability density functions (PDF) of the aspect ratio distributions of virions before (budding data; n = 22) and after scission (released data; n = 37) are shown. Normal fitting was done using MatLab (MathWorks, MA, USA). Fitting parameters were as follows: for budding data, mu = 0.35 ± 0.03 and sigma = 0.095 ± 0.026; for released data, mu = 0.37 ± 0.02 and sigma = 0.097 ± 0.013. Download [file mbo004162977sf3.tif]
